# Secondary arteriovenous malformation due to subclavian vein occlusion^☆^

**DOI:** 10.1016/j.radcr.2022.06.094

**Published:** 2022-07-29

**Authors:** Yukari Nakajima, Noriko Aramaki, Kazuo Kishi, Masashi Tamura, Seishi Nakatsuka, Masahiro Jinzaki, Masanori Inoue

**Affiliations:** a Department of Plastic and Reconstructive Surgery, Keio University School of Medicine, 35 Shinanomachi, Shinjuku-ward, Tokyo 160-8582, Japan; b Department of Radiology, Keio University School of Medicine, 35 Shinanomachi, Shinjuku-ward, Tokyo 160-8582, Japan

**Keywords:** Elephantiasis, NBCA, Embolization, Arteriovenous malformation, Extremities

## Abstract

An 80-year-old man underwent rectal resection and insertion of a central venous catheter through the left subclavian vein 16 years earlier. Following surgery, he developed edema of his left upper limb that became exacerbated and infected. Computed tomography showed occlusion of the subclavian vein and multiple arteriovenous shunts from the branches of the axillary artery to the venous sac of the axillary vein. Angiography confirmed numerous shunts between the branches of the axillary artery and vein and dilated collateral veins. Embolization of the venous sac was performed using coils, alcohol, and glue. Postprocedural angiography showed complete eradication of the nidus.

## Introduction

Thrombotic venous occlusion is a common complication associated with a central venous (CV) catheter. Most occlusions remain subclinical [1]; however, subsequent angiogenesis and the development of a symptomatic arteriovenous malformation (AVM) have been reported, but it is an extremely rare and poorly understood phenomenon [2,3]. The behavior of these AVMs is similar to that of congenital AVMs and can cause tissue edema, skin ulceration, and heart failure [2,3]. These AVMs should be treated based on the strategy for congenital AVMs.

An extremely rare case of elephantiasis due to an AVM caused by CV catheter-induced chronic subclavian venous thrombosis, which was treated successfully by transcatheter embolization, is described.

## Case report

An 80-year-old man presented with elephantiasis of the left arm. The patient underwent rectal cancer resection and CV catheter insertion through the left subclavian vein 16 years earlier. After the operation, he developed edema of the left arm, and it had continued to deteriorate.

**Fig. 1:**
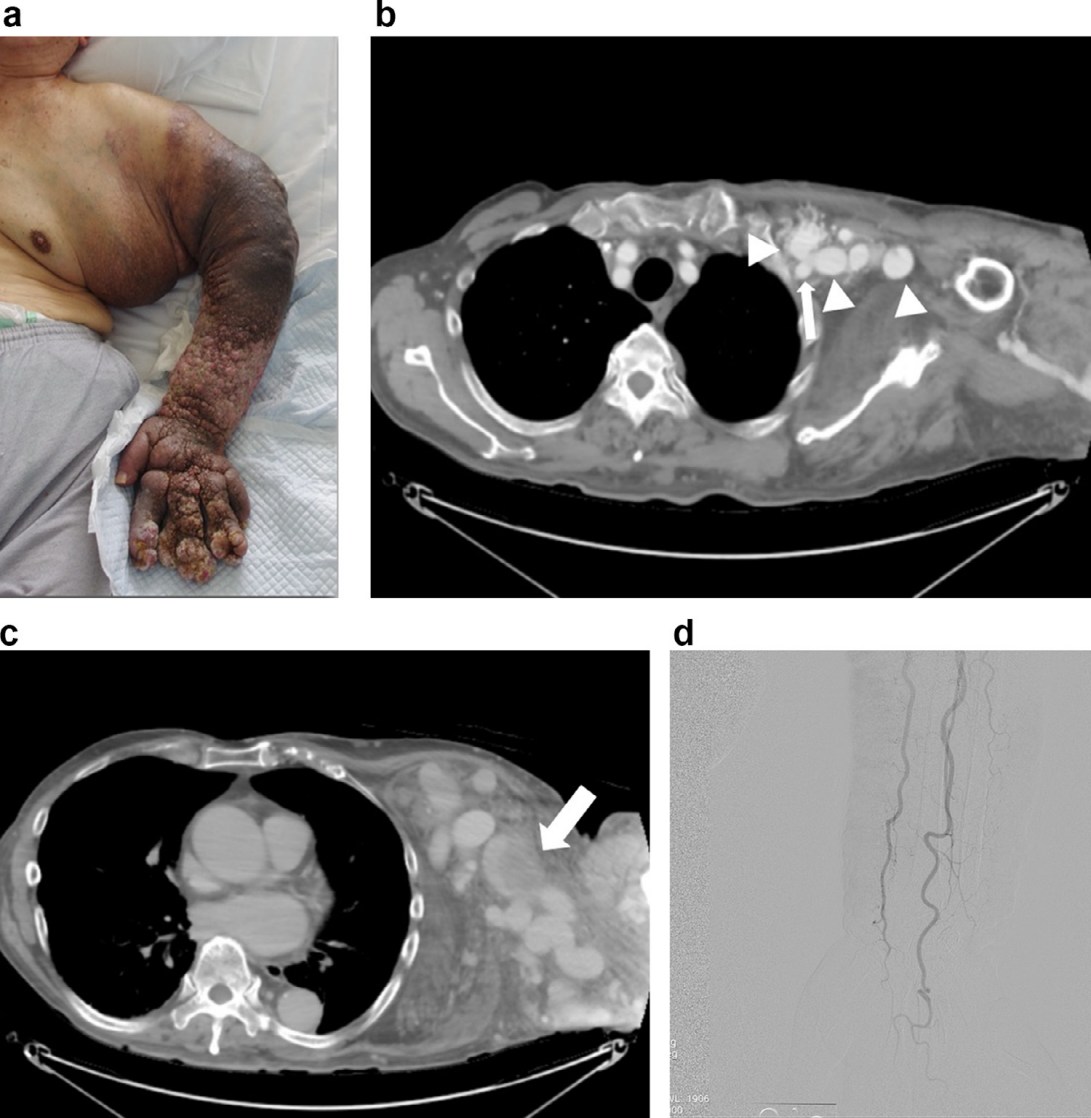
(A) Photograph of the left arm (pretreatment). The left arm has edema, cellulitis, lymphedema, pigmentation of the skin, and infection. (B, C) Preoperative contrast-enhanced CT. (B) Arterial phase. Collateral veins are dilated and enhanced. Arrow: axillary artery. Arrowhead: collateral veins. (C) Equilibrium phase. CT shows thrombus formation in a dilated collateral vein. (D) Digital subtraction angiography. The left arm has no lesion with vessels.

Thereafter, he had a long history of exacerbating edema, cellulitis, lymphedema, pigmentation of the skin, and infections of the left arm. Over the subsequent years, his symptoms worsened, and he was finally referred to our institution. He also had Parkinson's disease, dementia, and hyperuricemia. On physical examination, there was massive edema and serous fluid from his left chest to his fingers. The skin showed elephantiasis, and he had difficulty raising his arm (Fig. 1A).

Enhanced computed tomography (CT) showed occlusion of the left subclavian vein and numerous fine arterioles from the branches of the axillary artery shunting to dilated axillary veins with multiple dilated collateral veins. There was thrombosis of these dilated tortuous collateral veins around the arm and chest wall (Figs. 1B and C). On the other hand, there were no lesions with forearm vessels (Fig. 1D). There was also a thrombotic tendency on the preoperative blood test. The preoperative blood coagulation test results showed PT-INR 1.05, FDP-P 98.9 μg/mL, and D-dimer 46 μg/mL. Based on these findings, a type II AVM based on the Cho classification induced by thrombosis of the subclavian was suspected [4].

### Procedure

**Fig. 2:**
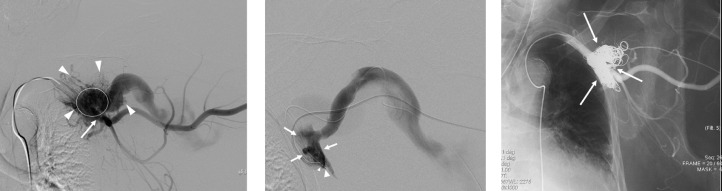
(A) DSA from the subclavian artery. Multiple feeding arteries (arrowhead) are visualized. Circle: initial venous sac. Arrow: subclavian artery. (B) DSA from the diagnostic catheter inserted from a drainage vein The tips of the diagnostic catheter (small arrowhead) and a microcatheter (large arrowhead) are located in the initial venous sac (arrow). (C) Angiography after embolization of the initial venous sac. The initial venous sac is embolized using coils (arrow), glue, and alcohol. Multiple feeding arteries have completely disappeared.

Embolization of the shunting point, namely the initial venous sac, was performed via a right femoral approach under general anesthesia. Digital subtraction angiography (DSA) from the subclavian artery and branches showed numerous abnormal vessels of the axillary artery shunted into the initial venous sac, which corresponded to the nidus of the AVM (Fig. 2A). There was efferent flow from the venous sac through the huge collateral veins to the chest wall (Fig. 2B). These findings corresponded with a type II AVM according to the angiographic classification [4].

One of the superficial drainage veins adjacent to the initial venous sac was directly punctured using 22-gauge and 18-gauge Chiba needles, followed by insertion of a microcathether and a 4-Fr angled diagnostic catheter, respectively.

Both catheters were advanced into the initial venous sac (Fig. 2C). Sac packing was performed through a 4-F diagnostic catheter using 0.035-inch fibered Interlock detachable coils (Boston Scientific Corp., Natick, MA) and fibered 0.035-inch pushable coils (Nester; Cook Medical Inc., Bloomington, IN). After stagnation of flow in the sac, 0.8 cc of 100% ethanol were injected, followed by injection of 0.5 ml of glue (N-butyl cyanoacrylate (NBCA) (Hystoacryl, B. Braun AG, Melsungen, Germany) mixed with iodized oil (Lipiodol; Andre Guerbet, Aulnay-Sous-Bois, France) at a ratio of 1:2 through the microcatheter. Postprocedural angiography showed complete eradication of the nidus (Fig. 2D).

### Outcome and follow-up

**Fig. 3:**
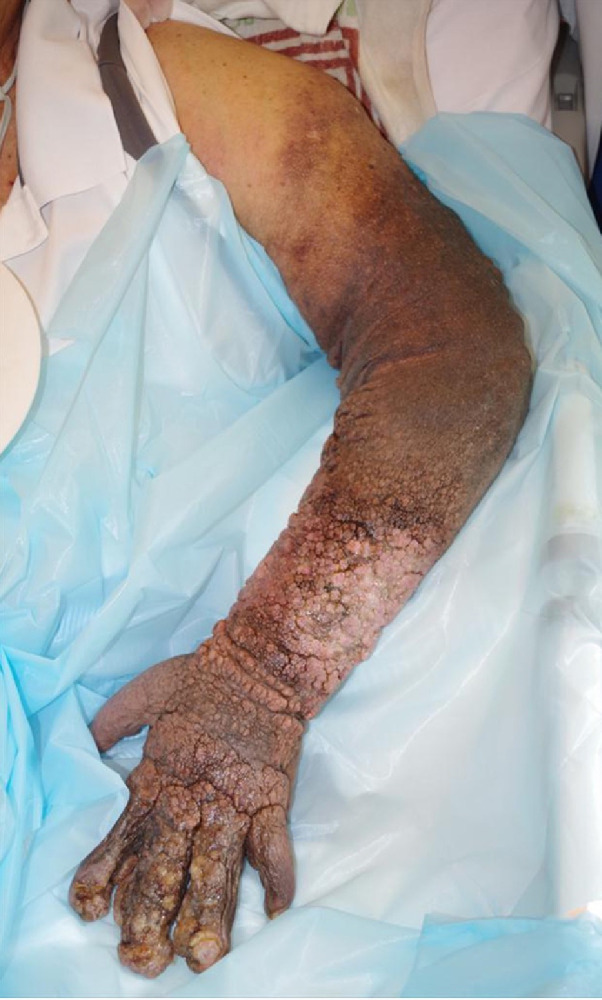
Photograph of the left arm (7 days after treatment). Edema of the left arm has improved remarkably.

The edema of the arm improved immediately after the procedure, and fluid exudation of the arm also decreased. Interdigital spaces could be identified, and elevation the left arm was possible 7 days after the treatment (Fig. 3). Contrast-enhanced CT showed complete occlusion of the nidus and thrombus formation in the drainage veins, with volume reduction of the drainage vessels around the neck and chest wall with no symptoms.

## Discussion

Though AVMs are commonly believed to be congenital lesions arising from aberrant vascular development during the intrauterine period, secondary AVMs following chronic venous sinus thrombosis are well known [5,6]. AVM formation in a peripheral region due to chronic venous thrombosis associated with central venous catheter insertion is rare [3,7], although an arteriovenous fistula other than an AVM is a rather well-known complication of iatrogenic venous catheter-induced complications [8].

It has been reported that AVM formation results from vasculogenesis, the differentiation of mesodermal precursors into endothelial cells, angiogenesis, and the formation of new vessels from preexisting ones [7]. Hypoxia and changes in shear stress or venous hypertension may result in upregulation of various kinds of growth factors [7] and stimulation of local angiogenesis. Finally, the precise pathogenesis of AVM formation due to chronic venous thrombosis remains unclear, but these changes in the local environment seem to lead to AVM formation [3,9].

Several case reports of AVM formation associated with venous thrombosis in the extremities and pelvis have been reported [2,3,7]. Lukies et al. reported a symptomatic case of acquired subclavian arteriovenous fistula formation due to long-term complications of chronic subclavian vein thrombosis, similar to the present case [3].

In the present case, the case was classified as stage III according to the Schobinger classification [10]. Treatment of AVMs is complex and should be reserved for symptomatic cases. Stage III and IV lesions should be treated because of the risk of progression, serious hemorrhage, and terminal cardiac failure.

Embolization and sclerotherapy are among the feasible treatment options and should be performed based on angiographic findings. Cho's classification is useful to predict the effectiveness of treatment, and this case was classified as a type II AVM, and eradication of the initial venous sac using embolic material and/or sclerosant is preferred. In a type II AVM, a transvenous approach or direct puncture of the initial venous sac is recommended [4]. In the present case, both direct puncture of an adjacent drainage vein and a transvenous approach were used because direct puncture of the initial venous sac was difficult. A microcatheter was inserted in addition to a 4-F diagnostic catheter, because the plan was to inject alcohol and glue through the microcatheter in combination with embolization using 0.035-inch coils through the 4-F diagnostic catheter.

Transcatheter embolization can improve symptoms and is superior to surgical resection from the perspective of functional preservation and lesser invasiveness in peripheral AVMs [11].

Lukies treated an AVM similar to the present case mainly by balloon venoplasty of the subclavian vein and stent graft insertion in the left subclavian artery [3]. In the present case, the subclavian vein was like a thread due to its long-term occlusion, and it seemed impossible and dangerous for it to be recanalized. Moreover, stent graft placement in the subclavian artery required dual antiplatelet therapy and has a risk of subclavian artery occlusion.

Link et al. described 2 cases of acquired lower limb AVMs due to chronic deep venous thrombosis [7]. In both cases, the AVM was treated by embolization of the feeding arteries using embolic materials including coils, alcohol, and glue. In their cases, the number of feeding arteries was not many; however, in the present case, multiple feeding arteries developed. Therefore, eradication of the initial venous sac was selected based on Cho's strategy [4]. Finally, a good outcome was achieved in the present case by occlusion of the nidus through a combination of direct puncture and a transvenous approach.

## Conclusion

A rare case of an AVM associated with long-term venous thrombosis due to CV catheter insertion was presented, and it suggests that some peripheral AVMs may develop secondary to chronic thrombosis. Generally, treatment of AVMs is challenging; however, the patient was treated successfully with embolization of the nidus.
